# The immune cell infiltrate in the tumour microenvironment of phaeochromocytomas and paragangliomas

**DOI:** 10.1530/ERC-22-0020

**Published:** 2022-08-16

**Authors:** N Tufton, R J Hearnden, D M Berney, W M Drake, L Parvanta, J P Chapple, S A Akker

**Affiliations:** 1 Centre for Endocrinology, Barts and the London School of Medicine and Dentistry, Queen Mary University of London, Charterhouse Square, London, UK; 2 Department of Endocrinology, St Bartholomew’s Hospital, Barts Health NHS Trust. West Smithfield, London, UK; 3 Department of Pathology, Royal London Hospital, Whitechapel, London, UK; 4 Department of Endocrine Surgery, St Bartholomew’s Hospital, Barts Health NHS Trust, West Smithfield, London, UK

**Keywords:** phaeochromocytoma, paraganglioma, immune cells, tumour microenvironment, macrophage, lymphocyte

## Abstract

Emerging evidence suggests the composition of the tumour microenvironment (TME) correlates with clinical outcome and that each tumour type has a unique TME including a variable population of inflammatory cells. We performed immunohistochemistry on 65 phaeochromocytoma and paraganglioma (PPGL) tumour samples with 20 normal adrenal medulla samples for comparison. The immune cells assessed were macrophages, lymphocytes and neutrophils, and we compared the proportion of infiltration of these immune cells with clinical and histopathological factors. There was a higher proportion of immune cells in tumour tissue compared to non-neoplastic adrenal medulla tissue, with a predominance of macrophages. There was a higher proportion of M2:M1 macrophages and T-helper lymphocytes in aggressive tumours compared to indolent ones. For *SDHB*-associated tumours, there was a higher proportion of M2 macrophage infiltration, with higher M2:M1 in aggressive *SDHB* PPGLs compared to indolent tumours. These data demonstrate that immune cells do infiltrate the TME of PPGLs, confirming that PPGLs are immunologically active tumours. Differences in the TME of PPGLs were observed between aggressive and indolent tumours. These differences could potentially be exploited as an aid in predicting tumour behaviour.

## Introduction

Phaeochromocytoma and paragangliomas (PPGLs) are rare neuroendocrine tumours of the autonomic nervous system that can arise anywhere from the skull base to the pelvic floor and may secrete catecholamines. All PPGLs should be considered as having metastatic potential (Lloyd *et al.* 2017), but, unlike other neoplasms, there are no known pathological or biochemical markers that can accurately predict which tumours have higher metastatic potential to be able to define risk stratification.

Up to 40% of PPGLs are associated with an underlying germline mutation, and these can be divided into two broad classifications: cluster 1, due to inappropriate activation of the hypoxia-angiogenesis pathway (*SDHA-D, AF2, MDH2, FH, vHL*, *EPAS1/*HIF2a and *PHD/EGLN*) and cluster 2 affecting kinase signalling pathways (*NF1, RET, TMEM127, MAX* and*KIF1B*) (Wachtel & Fishbein 2021). The different clusters have distinct phenotypes and secretory profiles (Eisenhofer *et al.* 2011). Recently, two further clusters have been added including a *Wnt*-altered subtype and cortical admixture subtype (Fishbein *et al.* 2017).

In addition to the tumour cells, solid tumours consist of a host of additional cells and an extracellular matrix (ECM) that together make up the tumour microenvironment (TME). Interest has grown in recent years in the potential role of the TME on tumour behaviour. Emerging evidence suggests the composition of the TME correlates with clinical outcome (Hendry *et al.* 2017) and that the TME of each cancer type is unique. Tumour evasion of immunosurveillance is a hallmark shared by all types of cancer (Hanahan & Weinberg 2011) and therefore the discovery of features acquired by tumours in response to the immune cells in their TME may open up new treatment strategies (Tamborero *et al.* 2018). Little is known about the TME of PPGLs. Compared to other tumours, PPGLs have a low rate of somatic mutations (Thorsson *et al.* 2018). This leads to the expectation that they have a low immunogenic antigen density and minimal inflammation (Farhat *et al.* 2019, Jimenez *et al.* 2020). However, patients with hormonally active PPGLs *in situ* have been shown to have raised systemic inflammatory markers (Zelinka *et al.* 2007, Bosanska *et al.* 2009).

Understanding the TME of PPGLs has the potential to provide valuable information about projected clinical outcomes, in turn guiding the intensity of post-operative surveillance strategies. It may also provide information that could lead to the development of novel therapies for metastatic disease, for which current options are limited and unsatisfactory. In this report, we discuss the immune cell infiltrate in the TME of PPGLs.

## Materials and methods

### Patients

This study was approved by Huntingdon Research Ethics Committee under the Genetics of Endocrine Tumours study (06/Q0104/133). Written consent was obtained from each patient.

Primary tumour samples were collected from patients with PPGLs that were operated on at Saint Bartholomew’s Hospital from 1994 to 2019. Clinical details of the patients included in this cohort are shown in Table 1. Non-neoplastic medulla was acquired from two separate sources; first, adjacent normal medulla was identified from the same specimens containing phaeochromocytoma tumour (*n* = 10) and secondly, non-neoplastic medulla was identified from patients that had undergone adrenalectomy for removal of an aldosterone-producing tumour (*n* = 10). For our cohort, tumours were defined as aggressive if metastases were present (*n* = 13) and/or the primary tumour showed evidence of local invasion into surrounding structures (*n* = 2). All other tumours were defined as indolent (*n* = 50), recognising that the indolent category will also include those PPGLs that have yet to demonstrate their aggressive potential.

**Table 1:** Clinical characteristics of patients from whom the PPGLs resected.

Total PPGLs	65
Total no. of patients	61
Gender	
Male (*n* (%))	43 (71%)
Female (*n* (%))	18 (29%)
Age at diagnosis (years) (mean (range))	40 (14–80)
Duration of FU (years) (mean (range))	7.8 (2–26)
Size of tumour (mm) (mean (range))	55.2 (10–235)
PCC (*n* = 38)	49.1 (10–114)
Abdo PGL (*n* = 17)	64 (19–235)
Thorax PGL (*n* = 5)	70.4 (45–117)
Pelvic PGL (*n* = 3)	41 (30–52)
HNPGL (*n* = 2)	70 (30–110)
No. of primary PPGLs (*n* (%))	56 (86%)
No. of multiple PGLs* (*n* (%))	4 (6%)
No. of recurrent PGLs (*n* ([%))	5 (8%)
Functional	
Yes	49 (75.3%)
No	8 (12.3%)
Unknown	8 (12.3%)
Raised MN > ULN (*n* (%))	21 (32%)
Raised NMA > ULN (*n* (%))	30 (46%)
Raised 3MT > ULN (*n* (%))	5 (2%)
Urine metanephrine levels (nmol) (n (%))	44 (67.7%)
Metanepherine (mean ± s.e.m.) NR < 2000)	11,122 ± 2840
Normetanepherine (mean ± s.e.m.) (NR < 4400)	22,760 ± 4930
3MT (mean ± s.e.m.) (NR < 2500)	2775 ± 478
Plasma metanephrine levels (pmol/L) (*n* (%))	23 (35.4%)
Metanepherine (mean ± s.e.m.) (NR < 510)	3475 ± 1175
Normetanepherine (mean ± s.e.m.) (NR < 1180)	9814 ± 2421
3MT (mean ± s.e.m.) (NR < 180)	155 ± 71.3
Aggressive (*n* (%))	15^+^ (23%)
Indolent (*n* (%))	50 (77%)
Genetics tested (*n* (%))	54 (83%)
Positive (*n* (%))	33 (50%)
Negative (*n* (%))	21 (32%)
*SDHB*	20 (8*) (40%)
*RET*	6 (9%)
*VHL*	4 (6%)
*SDHA*	2 (1*) (3%)
*FH*	1 (1.5%)
Negative	21 (2*) (32.3%)
Not tested (NT)	11 (3*) (20%)

*Denotes number of tumours in category that were aggressive in nature. ^+^Includes 13 PPGLs that were metastatic and 2 PPGLs that were locally invasive. FU, follow up; HNPGL, head and neck paraganglioma; MN, metanepherine; NMA, normetanepherine; NR, normal range; PCC, phaeochromocytoma; PGL, paraganglioma; 3MT 3-methyoxytyramine.

### Immunohistochemistry

All immunohistochemistry was performed on 3-μm paraffin-embedded tissue sections. Immunostains for immune cell markers were performed using the automated Ventana Discovery DAB Map 89 System (Ventana, Illkirch, France). Immune cell markers were used to identify different immune cells including macrophages (CD68+), T-cell lymphocytes (CD3+) and neutrophils (neutrophil elastase+), and then further analysis was undertaken to investigate subpopulations of macrophages: M1 macrophages (HLADR+), M2 macrophages (CD163+) and T-cell lymphocytes: T-helper cell lymphocytes (Th, CD4+), cytotoxic T-cell lymphocytes (Tc, CD8+). Antibodies and conditions used are outlined in Table 2. Haematoxylin and eosin (H&E) staining was undertaken to identify appropriate areas for scoring immune cell infiltrate. An experienced endocrine histopathologist (DB) reviewed all the slides and identified appropriate tumour areas (avoiding areas of necrosis) and/or areas containing normal medulla tissue for scoring. The Panoramic Scanner and Viewer Software (3DHISTECH, Budapest, Hungary) were used to scan and analyse stained slides. Immunopositive cells were counted in five different ‘hot spot’ high-power fields (HPF) using the software ImageJ (National Institutes of Health, USA). Data were expressed as a percentage of immunopositive immune cells relative to the total number of tumour cells per HPF. Proportions of immune cells were analysed by assessing hot spot areas to mirror the way slides are assessed in clinical practice. The selection of random areas may alter the findings. Preliminary data were analysed using tissue microarrays, but, with individual tumours having so much heterogeneity, we found that the results varied greatly between cores and were not reproducible. Ideally, dual staining for analysis of the macrophage and T-cell subpopulations could has been undertaken to overcome this, but we found dual staining with immunoflurescence demonstrated high background auto-fluorescence making results difficult to interpret.

**Table 2:** Primary antibodies for immunohistochemistry.

Primary antibody	Manufacturer	Catalogue number	Species	Dilution IHC
CD68	DAKO	IR613	Mouse	1:2
CD3	DAKO	A0452	Rabbit	1:300
Neutrophil elastase	Abcam	Ab68672	Rabbit	1:100
CD163	Abcam	Ab74604	Mouse	1:500
HLA-DR (TAL 1B5)	Abcam	Ab20181 M4	Mouse	1:100
CD4	Abcam	Ab133616	Rabbit	1:100
CD8	DAKO	160621	Mouse	1:100

### Statistical analysis

GraphPad Prism version 9.0 was used for statistical analysis. Kolmogorov–Smirnov test was used to check for normality of data distribution. Equality of variances was tested using the Brown–Forsythe test for equal variances. For parametric data with equal variances, Student independent t-tests and one-way ANOVA tests (with Tukey’s multiple comparison test) were used for data analysis. For parametric data with unequal variances, Welch’s unequal variances t-tests and Welch’s one-way ANOVA (with Dunnett’s T3 multiple comparison test) were used. For non-parametric data, chi-square, Mann–Whitney U and Kruskal–Wallis tests (Dunn’s multiple comparison tests) were used for data analysis. Pearson’s r test was used to analyse correlations. A *P* value cut-off of <0.05 was used for assessing statistical significance.

## Results

**Figure 1:**
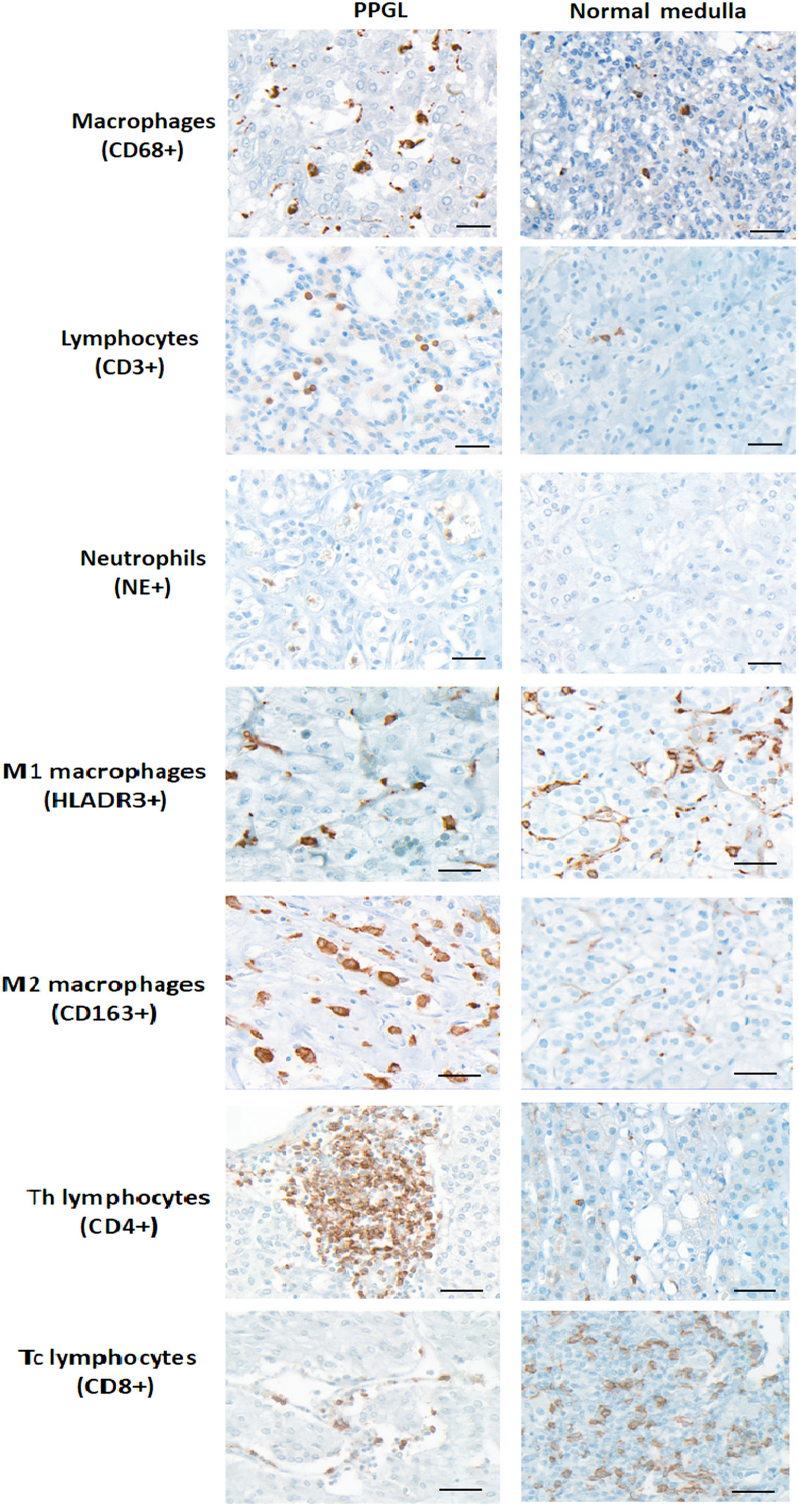
Representative images of PPGLs and non-neoplastic medulla immunohistochemical staining for immune cell markers. Formalin-fixed, paraffin-embedded tissue sections were immunolabelled with CD68+ (macrophages), CD3+ T-cell lymphocytes), neutrophil elastase+ (NE, neutrophils), HLADR+ (M1 macrophages), CD163+ (M2 macrophages), CD4+ (Th T-helper lymphocytes) and CD8+ (Tc cytotoxic T lymphocytes). Scale bar = 40 μm.

The immune cell infiltrate was analysed by immunohistochemistry for 65 tumour samples and 20 non-neoplastic adrenal medulla samples. The immune cells assessed were macrophages, T-cell lymphocytes and neutrophils. Clinical details of the patients included in this cohort are shown in Table 1. The cohort consisted of 71% male patients with tumour samples from the adrenal medulla (58.5%) and extra-adrenal abdominal PGLs (26%), with a mean follow-up duration of 7.82 years (2–26years). Examples of staining for the different antibodies are shown in Fig. 1.

**Figure 2:**
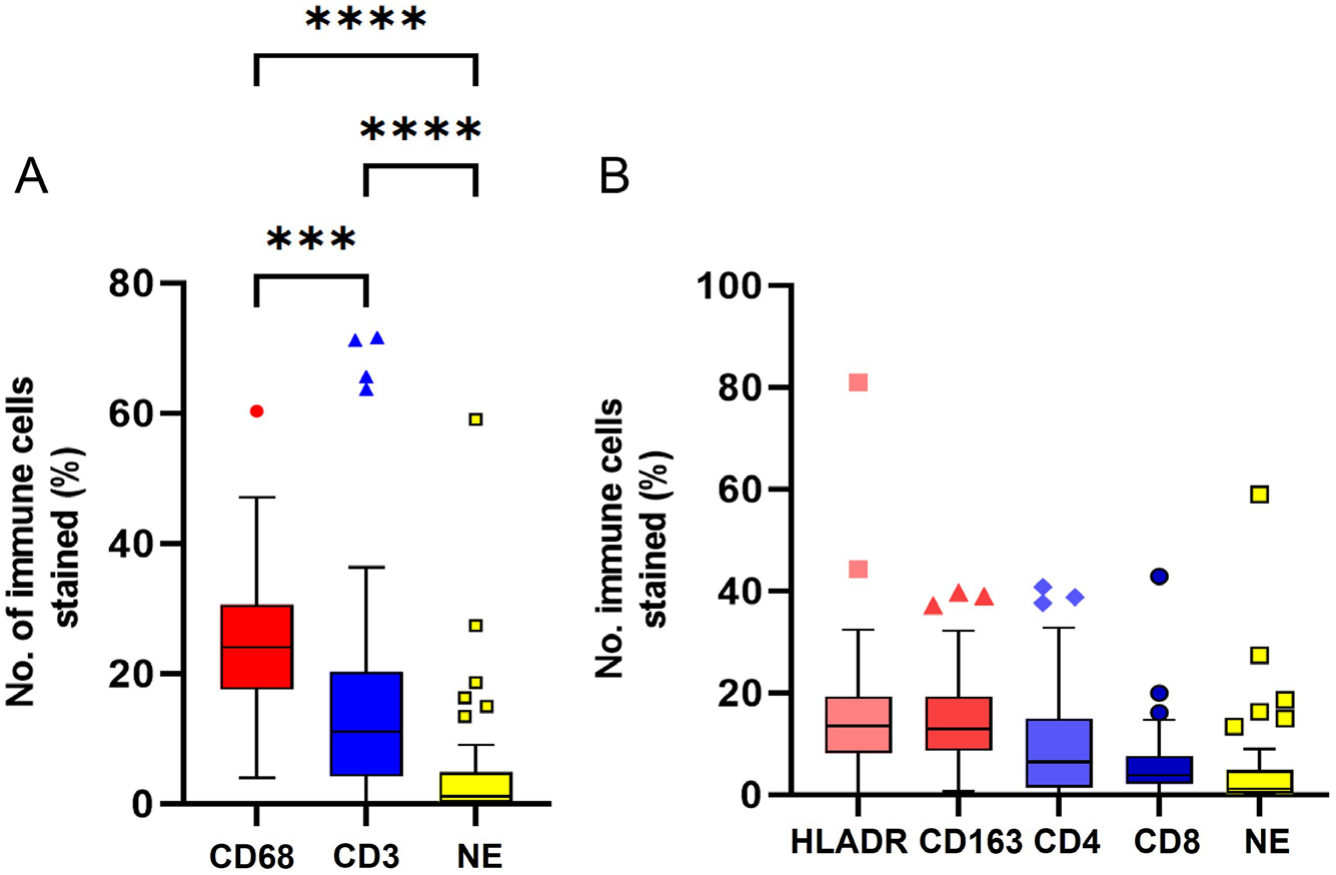
Immunohistochemical analysis of immune cells in PPGL tumours. Immune cells analysed: macrophages (CD68+, M1 HLADR+, M2 CD163+), lymphocytes (CD3+, Th CD4+, Tc CD8+) and neutrophils (NE, neutrophil elastase+). Box-and-whisker plots of mean, IQR and outlying points of the percentage of immunopositive cells as a proportion of the total number of cells for PPGL tumour tissue (*n* = 65). (A) Comparison between major immune cell types. Statistical analysis by ANOVA with Tukey’s multi-comparison tests (*P* < 0.001). Macrophages vs lymphocytes (*P* = 0.0002), macrophages vs neutrophils (*P* < 0.001), lymphocyte vs neutrophils (*P* < 0.001). (B) Subsets of macrophages (HLADR+, CD163+) and lymphocytes (CD4+, CD8+). ANOVA with Tukey’s multi-comparison tests (*P* < 0.001). Th, T-cell helper lymphocyte, Tc, T-cell cytotoxic lymphocytes. A full colour version of this figure is available at https://doi.org/10.1530/ERC-22-0020.

**Figure 3:**
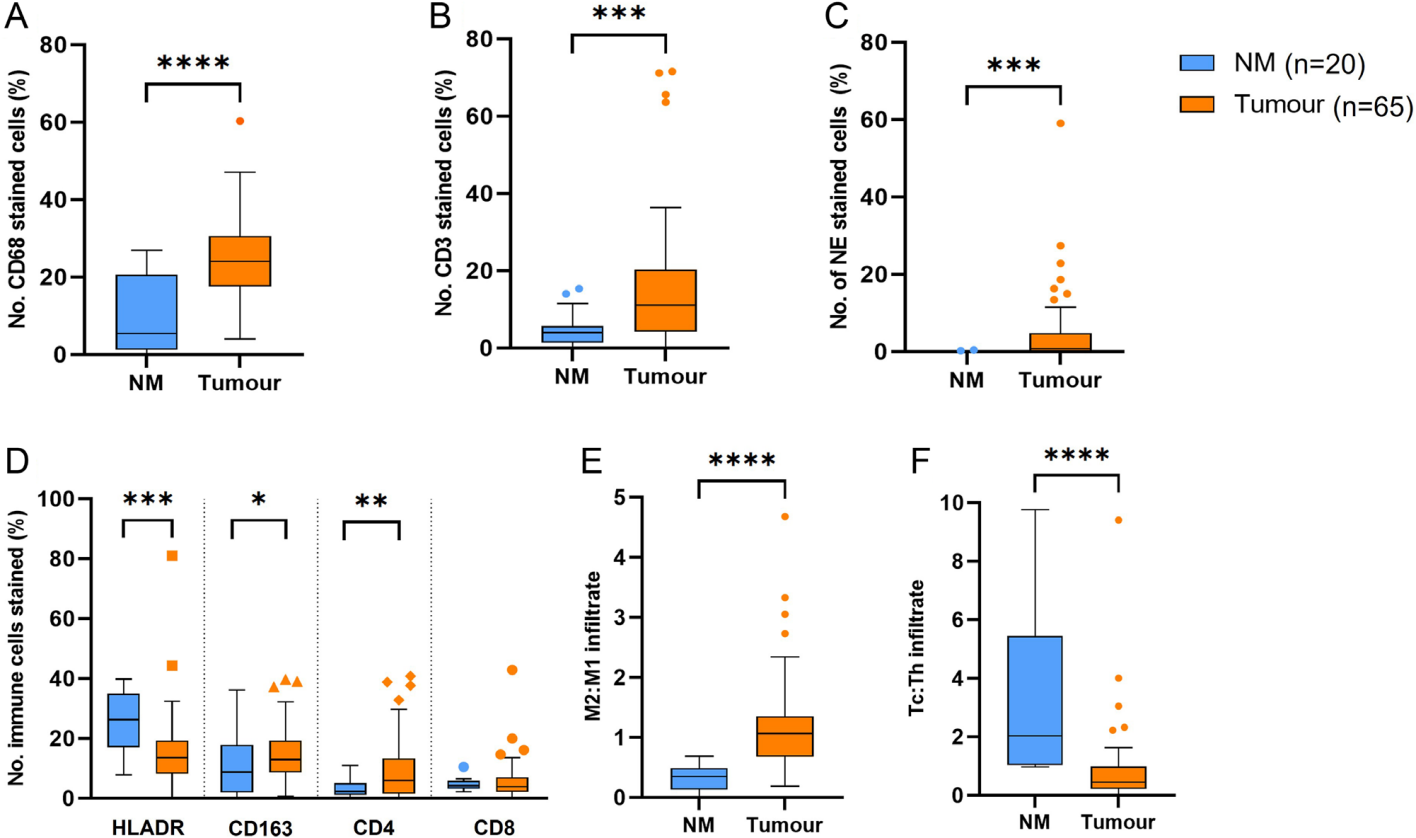
Immunohistochemical analysis of immune cells in PPGL tumours compared to non-neoplastic adrenal medulla. Immune cells analysed: (A) macrophages (CD68+), (B) lymphocytes (CD3+), (C) neutrophils (NE, neutrophil elastase+), (D and E) M1 (HLADR3), M2 (CD163+), (D and E) T-helper lymphocytes (CD4+), Cytotoxic T cells (CD8+). Box-and-whisker plots of mean, IQR and outlying points of the percentage of immunopositive cells as a proportion of the total number of cells for PPGL tumour tissue (*n* = 65) and non-neoplastic adrenal medulla tissue (NM *n* = 20). (A) CD68 normal distributed data (****P < 0.0001) (Student two-tailed t-test), (B) lymphocytes (*P* = 0.0003). (C) Neutrophils (*P* = 0.0004) are non-parametric data Mann–Whitney U test (***P < 0.001). (D) Subset of macrophages: HLADR3 (*P* = 0.0008), CD163 (*P* = 0.0259), CD4 (*P* = 0.0015) and CD8 (*P* = 0.4). (E) M2:M1 (*P* < 0.001). (F) Tc:Th (*P* = 0.0019). A full colour version of this figure is available at https://doi.org/10.1530/ERC-22-0020.

There was a significant proportion of immune cells in the TME of PPGLs, with a prominence of macrophages, and very few neutrophils (Fig. 2). There was a higher proportion of immune cells in tumour compared to non-neoplastic adrenal medulla tissue (Fig. 3) across all of the immune cell types. There were higher proportions of CD4 T lymphocytes (Th) compared to CD8 T lymphocytes (Tc) in the tumour tissue (*P* = 0.0006). There was no difference in absolute numbers between the subpopulations of macrophages (HLADR vs CD163, *P* = 0.77).

**Figure 4:**
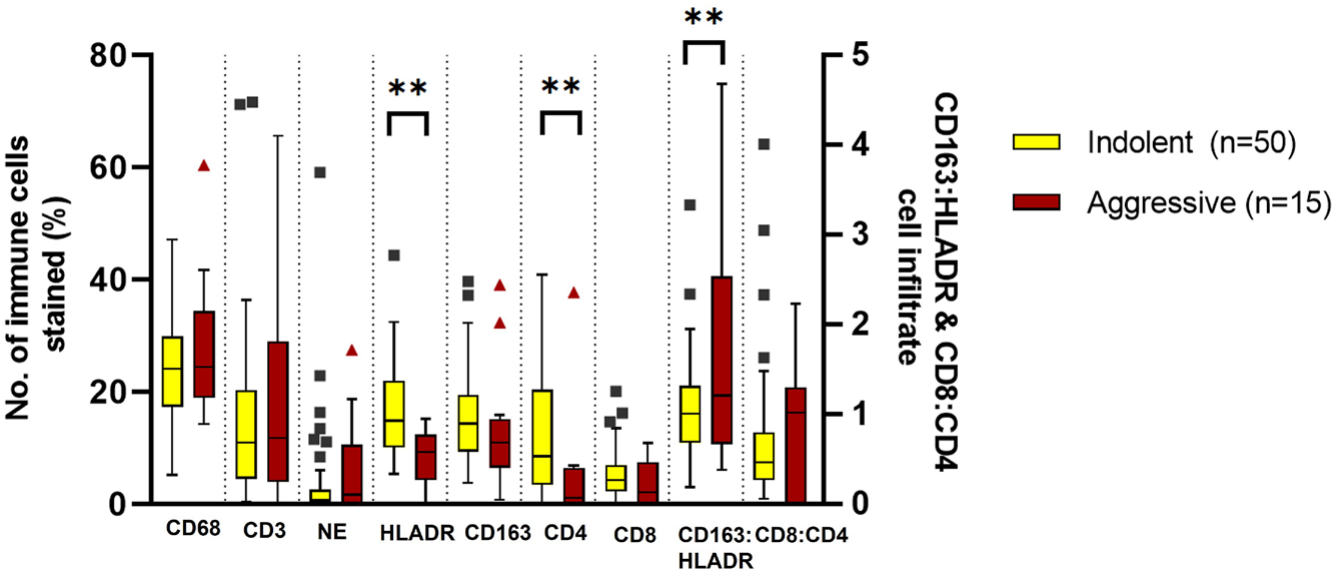
Immunohistochemical analysis of immune cells in indolent and aggressive PPGL tumours. Immune cells analysed: macrophages (CD68+), lymphocytes (CD3+), neutrophils (NE, neutrophil elastase+), M1 (HLADR3), M2 (CD163+), T-helper lymphocytes (CD4+) and cytotoxic T cells (CD8+). Box-and-whisker plot of mean, IQR and outlying points of the percentage of immunopositive cells as a proportion of the total number of cells for indolent tumours (*n* = 50), aggressive tumours (*n* = 15). **P < 0.01 (Mann–Whitney U test. M1 macrophages (*P* = 0.0001), CD4 lymphocytes (*P* = 0.0039), M2:M1 (*P* = 0.01). Non-significant results of indolent vs aggressive tumour tissue CD68 *P* = 0.358, CD3 *P* = 0.999, NE *P* = 0.53, CD163 *P* = 0.138, CD8 *P* = 0.24 and Tc:Th *P* = 0.893. A full colour version of this figure is available at https://doi.org/10.1530/ERC-22-0020.

There were no differences in overall immune cell infiltration of macrophages, T cells or neutrophils between indolent and aggressive tumours. However, there was a lower proportion of HLADR (M1) macrophages (*P* = 0.0001) and a higher proportion of CD4 T-helper lymphocytes (*P* = 0.0039) in aggressive tumours with a higher M2:M1 ratio (*P* = 0.01) (Fig. 4). These differences were maintained when the data were analysed with the two locally invasive tumours removed from the aggressive group.

**Figure 5:**
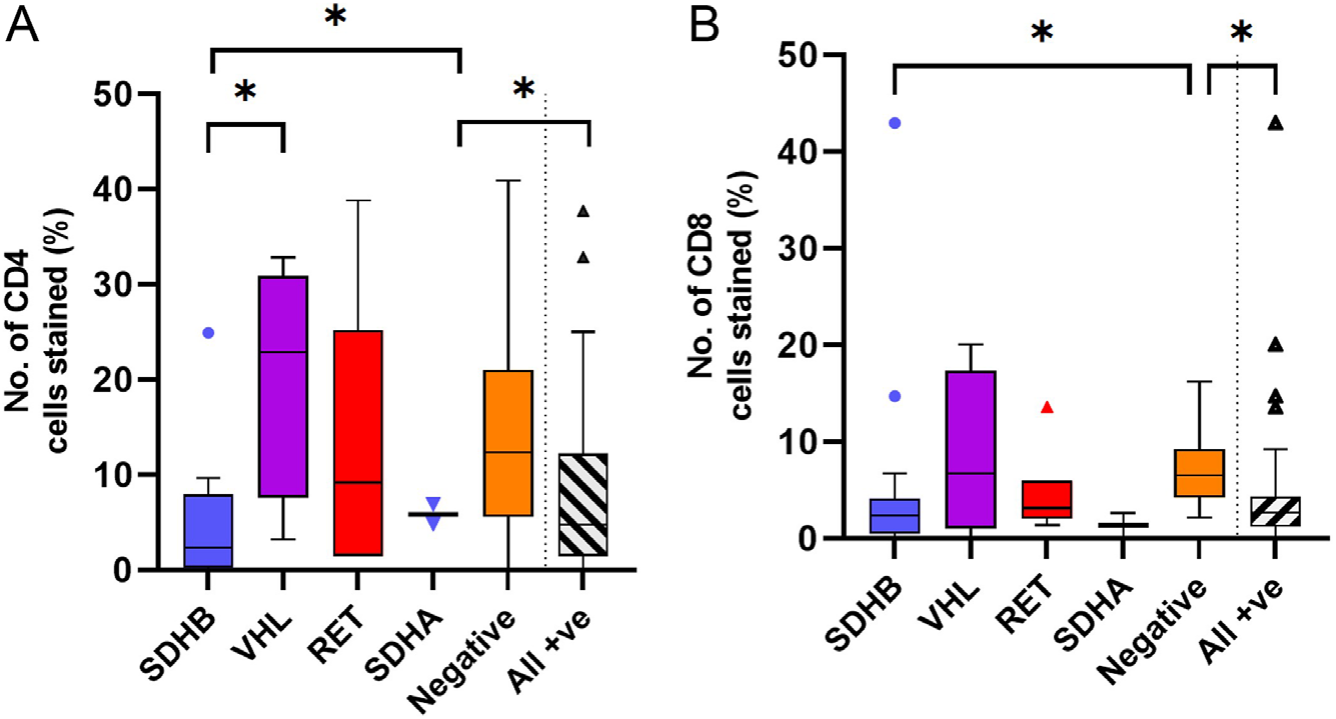
Immunohistochemical analysis of immune cells in PPGL tumours divided into genetic groups. Immune cells analysed: macrophages (CD68+), lymphocytes (CD3+), neutrophils (NE, neutrophil elastase+), M2:M1 (CD163+:HLADR+) and T-helper lymphocytes:Cytotoxic T cells (Tc:Th, CD4+:CD8+). (A) Box-and-whisker plot of mean, IQR and outlying points of the percentage of immunopositive cells as a proportion of the total number of cells for the different underlying genetic mutations for number of T-helper lymphocytes (CD4+). Statistical analysis by Kruskal–-allis test with Dunn’s multiple comparison tests (*P* = 0.0315*), *SDHB vs VHL* (*P* = 0.0211*), *SDHB* vs negative (*P* = 0.0427*), all positive vs negative (*P* = 0.027*). (B) Box-and-whisker plot of mean, IQR and outlying points of the percentage of immunopositive cells as a proportion of the total number of cells for the different underlying genetic mutations for number of cytotoxic T lymphocytes (CD8+). Statistical analysis by Kruskal–Wallis test with Dunn’s multiple comparison tests (*P* = 0.0185), *SDHB* vs negative (*P* = 0.033*), all positive vs negative (*P* = 0.00473*). A full colour version of this figure is available at https://doi.org/10.1530/ERC-22-0020.

**Figure 6:**
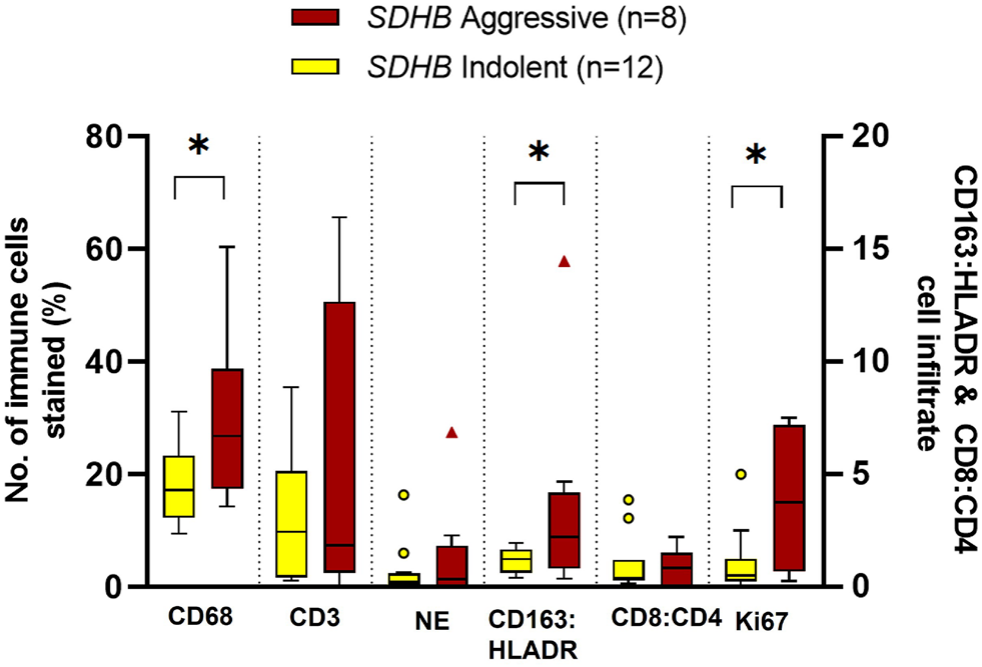
Immunohistochemical analysis of immune cells in PPGL tumours that arose in patients with *SDHB* mutations. Immune cells analysed: macrophages (CD68+), lymphocytes (CD3+), neutrophils (NE, neutrophil elastase+), M2 (CD163+):M1 (HLADR+) macrophages, Tc (CD8+):Th (CD4+) lymphocytes and Ki67 index. Box-and-whisker plot of mean, IQR and outlying points of the percentage of immunopositive cells as a proportion of the total number of cells of immune cell infiltration in indolent (*n* = 12) and aggressive (*n* = 8) PPGLs in patients with *SDHB* mutations. Macrophages (*P* = 0.029) (Student t-test), lymphocytes (*P* = 0.45) (Welch’s t-test), neutrophils (*P* = 0.955) (Mann–Whitney U test), M2:M1 (*P* = 0.02) (Mann–Whitney U test), CD8:CD4 (*P* = 0.868) (Mann–Whitney U test) and Ki67 (*P* = 0.03) (Welch’s t-test). A full colour version of this figure is available at https://doi.org/10.1530/ERC-22-0020.

There was no difference in immune cell infiltration associated with the location of the tumour and very few differences between underlying genetic diagnosis. There was a higher M2:M1 ratio in cluster 1 (*SDHx, VHL, FH*, *n* = 27) tumours compared to cluster 2 (*RET*, *n* = 6) (*P* = 0.017) and a lower proportion of CD8 cytotoxic (Tc) lymphocytes in PPGLs with a known genetic diagnosis (*P* = 0.047). There were significantly higher levels of CD4 Th lymphocytes in *VHL* tumours compared to *SDHB* tumours and the lowest levels of both Th and Tc lymphocytes were observed in PPGLs with underlying *SDHB* mutations (Fig. 5). Specifically, within the *SDHB* tumours there was a higher proportion of CD163 expressing macrophages (M2, *P* = 0.02) in aggressive *SDHB* tumours, correlating with a higher Ki67 index (Fig. 6). No differences were observed within the other genetic subgroups.

No correlations were identified between the tumour size and Ki67 index or immune cell infiltration, although there was a trend towards increasing macrophage infiltration with a higher Ki67 index (*P* = 0.07) and increasing tumour size (*P* = 0.08).

There was a positive correlation between tumour size and plasma metanephrine (MN, *P* = 0.016) and normetanephrine (NMN, *P* = 0.001) concentrations. With regards to immune cell infiltration and functional status of the tumours, there was a positive correlation between neutrophil infiltration and MN levels (*P* = 0.004) and between macrophage infiltration and NMN levels (*P* = 0.02).

## Discussion

Understanding the importance of inflammatory cells in PPGLs has two important clinical implications. Most obviously, if this component of the TME has a role in the pathogenesis of more aggressive tumour phenotypes, then greater understanding may open up potential therapeutic interventions targeting this. A more immediate implication would be to help define those PPGLs with a more aggressive potential that may benefit from closer post-operative surveillance.

These data demonstrate that the immune cell component of the microenvironment is markedly different in PPGLs compared to the normal adrenal medulla, with the most striking feature being the higher proportion of macrophages in the TME. As in previous studies, macrophages were the abundant immune cell type (Gao *et al.* 2020, Guadagno *et al.* 2020, Batchu 2021), with a relative T-cell lymphocyte depletion and very low levels of neutrophils. The analysis shows that the TME of PPGLs is dominated by CD163-expressing macrophages (M2), whereas normal medulla ECM predominately contains HLADR-expressing macrophages (M1). M2 macrophages, also known as tumour-associated macrophages, are anti-inflammatory and immunosuppressive and, as with other solid tumours (Ryder *et al.* 2008, Hao *et al.* 2012, Lan *et al.* 2013, Fang *et al.* 2014, Wei *et al.* 2014, Ruffell & Coussens 2015, Carron *et al.* 2017, Mantovani *et al.* 2017, Maolake *et al.* 2017), may be associated with cell proliferation and tumour progression in PPGLs, possibly via immune evasion. We observed large variations in immune cell infiltration between samples as reported in other cohorts (Farhat *et al.* 2019, Gao *et al.* 2020, Batchu 2021), demonstrating the wide heterogeneity that exists within these tumours. Even with this heterogeneity, however, we were able to demonstrate some significant differences in the TME of different types of PPGLs.

Generally, there were low levels of T cells throughout the PPGLs as previously reported (Tamborero *et al.* 2018, Thorsson *et al.* 2018, Farhat *et al.* 2019). The observed T-cell depletion may be a catecholamine effect, as catecholamines have been reported to suppress T lymphocytes Th1, Th2, cytotoxic and natural killer cells (Madden *et al.* 1995, Nasi *et al.* 2019) and exert inhibitory effects on T-cell proliferation due to chronic stress in mice (Edgar *et al.* 2003). However, we observed the highest levels of T cells in adrenaline-producing PPGLs, which are generally associated with a benign/indolent course of disease. Gao and coworkers reported a positive correlation between CD8 Tc cells and plasma adrenaline levels and a negative correlation with grading of adrenal phaeochromoctyoma and paraganglioma (GAPP) score (Gao *et al.* 2020). Catecholamines have been reported to suppress lymphocyte action (Madden *et al.* 1995, Edgar *et al.* 2003, Nasi *et al.* 2019), but these findings suggest that this may be an effect of noradrenaline rather than adrenaline. The data suggest that hormone production by the tumour may influence the immune cell infiltration into the TME and it is possible that this may have an influence on tumour behaviour as recently shown for glucocorticoids in adrenocortical carcinoma (ACC) (Landwehr *et al.* 2020).

The question had previously been raised whether tumours arising at different locations in the body had different TMEs (Balkwill *et al.* 2012). Although individual sample sizes are small for each location, we observed no differences in immune cell profiles across the different locations of the tumours, consistent with other recent reports (Farhat *et al.* 2019, Batchu 2021).

Very few differences were observed in immune cell infiltration between PPGLs caused by different underlying mutations, similar to previous studies (Farhat *et al.* 2019), perhaps reflecting the wide clinical heterogeneity within each genetic group with regards to tumour behaviour. The only difference across all genotypes was a lower proportion of T-cytotoxic lymphocytes, with the lowest level observed in PPGLs associated with *SDHB* mutations. Cytotoxic T cells are usually associated with a good clinical outcome as they have the ability to kill tumour cells. *SDHB* tumours are known to have a greater metastatic potential and the lower levels observed in these tumours correlate with this behaviour. This suggests that there may be inhibition of either recruitment or action of these immune cells, in turn allowing a greater chance of proliferation and malignant transformation, due to immune system evasion.

Specifically with the *SDHB*-associated PPGLs, in addition to higher Ki67 scores and lower cytotoxic T-cell proportion, there was also a higher infiltration of macrophages, with M2 macrophages dominating in aggressive *SDHB* tumours compared to indolent ones. Another study performed an analysis of tissue microarray (TMA) cores of 48 PCCs and 4 PGLs and found the highest proportion of M2 macrophages occurred in 3 out of 4 of their *SDHx t*umours (Farhat *et al.* 2019). These histological tumour markers could potentially be used, in addition to clinical factors (tumour size, etc), to aid in predicting which of these tumours have the highest metastatic potential, thereby allowing surveillance to be tailored to individual risk.

Higher proportions of immune cells were found in both indolent and aggressive tumours, but subset analysis of macrophages and lymphocytes demonstrated differences between these tumour groups. There was a higher proportion of HLADR-expressing macrophages (M1) and CD4 Th cells in indolent tumours and conversely a higher M2:M1 ratio in aggressive tumours. M1 macrophages are pro-inflammatory and therefore can potentially lead to tumour rejection (Viola *et al.* 2019, Liu *et al.* 2021), which may be a mechanism for why these tumours do not become aggressive and metastasise. M2 macrophages, in contrast, are anti-inflammatory, aiding evasion of the immune system (Liu *et al.* 2021) potentially allowing these tumours to proliferate and disseminate. Not surprisingly, given the higher rates of metastases that are seen in patients with underlying *SDHB* mutations, there is a bias within our cohort with 8 out of the 15 aggressive tumours harbouring an underlying *SDHB* mutation. Our most significant results were seen within the *SDHB*-associated tumours, although the significance remained when analysing the group as a whole. These data may provide interesting insight specifically into the TME of the *SDHB*-associated PPGLs, as even within this small group, differences were observed between the aggressive and indolent tumours demonstrating the heterogenicity within, as well as between, groups.

Could these observations be exploited to identify those PPGLs within the indolent group that may have more aggressive potential? We selected the 5 tumours from the indolent cohort with the highest M2:M1 ratios and looked for other factors associated with aggressiveness; 3 of these also had the highest Ki67 scores, 3 had *SDHB* mutations and 4 out of the 5 were larger than 40 mm. This preliminary data suggest that the TME is worthy of further definition and study. If these data are validated in a larger tumour set, the TME may be an additional factor that could be included in scoring systems such as those most recently proposed (Wang *et al.* 2020, Yamazaki *et al.* 2020) to aid in better stratification of patients at higher risk.

As in other PPGL studies, this study has limitations due to the relatively small sample size and the known heterogeneity between different PPGL tumours and within individual PPGL tumours. With the small sample size, subset analysis should be interpreted with caution. As discussed in ‘Methods’, there are also different approaches for assessing proportions of immune cells which may influence results and the M1/M2 classification of macrophages may be considered an oversimplification of the true situation, as these cells are highly plastic and adaptable (Viola *et al.* 2019).

Overall, however, these data provide preliminary support for the two important clinical benefits we outlined earlier where analysis of the inflammatory cells may provide a useful marker of potential aggressiveness, and if further work provides a greater understanding of the TME, this has the potential to allow more targeted treatment of aggressive PPGLs.

## Declaration of interest

The authors declare that there is no conflict of interest that could be perceived as prejudicing the impartiality of the research reported.

## Funding

N T is funded by The Medical College of Saint Bartholomew’s Hospital Trust (registered charity number 1115519). The Microscopy core is funded by CRUK microscopy core service grant at Barts Cancer Institute (Core Award C16420/A18066).

## Author contribution statement

N T, S A A, R J H and J P C were involved in study design and data interpretation. N T drafted the manuscript and S A A, J P C, W M D, L P, R H and D M B were involved in reviewing and revising the manuscript. All authors approved the final version.
